# Binary Gene Expression Patterning of the Molt Cycle: The Case of Chitin Metabolism

**DOI:** 10.1371/journal.pone.0122602

**Published:** 2015-04-28

**Authors:** Shai Abehsera, Lilah Glazer, Jenny Tynyakov, Inbar Plaschkes, Vered Chalifa-Caspi, Isam Khalaila, Eliahu D. Aflalo, Amir Sagi

**Affiliations:** 1 Department of Life Sciences, Ben-Gurion University of the Negev, Beer-Sheva, Israel; 2 The National Institute for Biotechnology in the Negev, Ben-Gurion University of the Negev, Beer-Sheva, Israel; 3 Department of Biotechnology Engineering, Ben-Gurion University of the Negev, Beer-Sheva, Israel; Kansas State University, UNITED STATES

## Abstract

In crustaceans, like all arthropods, growth is accompanied by a molting cycle. This cycle comprises major physiological events in which mineralized chitinous structures are built and degraded. These events are in turn governed by genes whose patterns of expression are presumably linked to the molting cycle. To study these genes we performed next generation sequencing and constructed a molt-related transcriptomic library from two exoskeletal-forming tissues of the crayfish *Cherax quadricarinatus*, namely the gastrolith and the mandible cuticle-forming epithelium. To simplify the study of such a complex process as molting, a novel approach, binary patterning of gene expression, was employed. This approach revealed that key genes involved in the synthesis and breakdown of chitin exhibit a molt-related pattern in the gastrolith-forming epithelium. On the other hand, the same genes in the mandible cuticle-forming epithelium showed a molt-independent pattern of expression. Genes related to the metabolism of glucosamine-6-phosphate, a chitin precursor synthesized from simple sugars, showed a molt-related pattern of expression in both tissues. The binary patterning approach unfolds typical patterns of gene expression during the molt cycle of a crustacean. The use of such a simplifying integrative tool for assessing gene patterning seems appropriate for the study of complex biological processes.

## Introduction

Crustaceans possess a rigid mineralized exoskeleton consisting of a layered cuticle that covers the soft body parts to provide support and protection [1]. Growth in crustaceans demands periodic shedding and replacement of the cuticle in a cyclical process called molting. The molting cycle can be divided into four major stages, inter-molt, pre-molt, ecdysis, and post-molt, by order of occurrence [2]. Inter-molt is the stage when the animal is between two molting events and is characterized by a fully developed cuticle [3]. Inter-molt is followed by pre-molt, during which time the existing cuticle is partly digested, de-mineralized and re-absorbed; the creation of a new non-mineralized cuticle occurs simultaneously. At ecdysis, corresponding to the most obvious manifestation of the molting cycle, the old exoskeleton is shed. At post-molt, water uptake allows the new flexible cuticle to stretch to its full size, while mineralization leads to cuticle hardening [4, 5]. Molting, therefore, necessitates a bi-directional movement of minerals, mostly calcium carbonate, into the cuticle during post-molt and out of the cuticle during pre-molt [6]. Accordingly, distinct physiological processes characterize the different molting stages.

The freshwater crayfish *Cherax quadricarinatus*, the model organism of this study, inhabits a low calcium environment. As such, special adaptations are required for these animals to obtain sufficient amounts of calcium during molting. Gastroliths are transient calcium storage organs located on both sides of the stomach wall that undergo mineralization during pre-molt and collapse into the stomach for full digestion during post-molt, thereby providing calcium for cuticular mineralization [7–9]. Different temporal patterns of mineralization are thus revealed upon comparison of the cuticle and gastrolith [10].

In the crayfish cuticle and gastrolith, deposited minerals and various proteins are closely associated with chitin fibers [11, 12]. Specifically, chitin, a linear homopolymer of β 1–4 linked N-acetylglucosamine residues, functions as a fibrous scaffold for exoskeletal formation [13, 14]. As a major component of all crustacean exoskeletal scaffolds, chitin metabolism is closely associated with the molting cycle [15, 16]. In the whiteleg shrimp *Litopenaeus vannamei*, transcripts of genes encoding the enzymes chitin synthase and chitinase presented varying pattern of expression in several different tissues during the molt cycle, reflecting the need of the shrimp to either synthesize or hydrolyze exoskeletal chitin [17]. In the crayfish *C*. *quadricarinatus*, the chitin deacetylase gene is up-regulated during pre-molt when compared to its levels in the hypodermis and the gastrolith during inter-molt [3]. Precursors for chitin synthesis used in the assembly of the new cuticle also differed over the course of the molting cycle. N-acetyl glucosamine-6-phosphate, a chitin breakdown product derived from the old cuticle, was primarily used for chitin synthesis during pre-molt, while during post-molt, diet-derived glucose served as the main chitin precursor [16, 18].

Using next-generation sequencing, molt cycle-related transcriptomic library construction and real time quantification of gene expression, we have developed a novel binary patterning study approach. The strength of binary patterning lies in its ability to provide a simplified integrative view of complex biological processes. Using our approach, the molt related patterns in which chitin synthesis, degradation, utilization and recycling are exhibited, were revealed. It demonstrates the interconnecting relationships between mineralization patterns and expression patterns of chitin metabolism at each tissue, with respect to molt stage related structural and energetic processes.

## Materials and Methods

### Molt induction and sample preparation

The broodstock for the *C*. *quadricarinatus* currently being cultured in Israeli aquafarms was imported into Israel from Australia approximately three decades ago. *C*. *quadricarinatus* males were grown in and collected from artificial ponds at Ben-Gurion University of the Negev, Beer-Sheva, Israel. Food comprising shrimp pellets (Rangen Inc., Buhl, ID, USA, 30% protein) was supplied *ad libitum* three times a week. Temperature was kept at 27 ± 2°C, and a photoperiod of 14 h light and 10 h dark was applied. Water quality was assured by circulating the entire volume of water through a bio-filter. The pH of the water was 8.3 ± 0.5, the nitrite concentration was less than 0.1 mg/l, the nitrate concentration was less than 50 mg/l, ammonium levels were negligible, and oxygen levels exceeded 5 mg/1. Animals were anesthetized in ice-cold water prior to dissection. Inter-molt crayfish were held in individual cages and endocrinologically induced to enter pre-molt through daily α-ecdysone injection as described [19]. For all dissection procedures, crayfish were placed on ice for 10–15 min until anesthetized. Two exoskeletal-forming tissues were dissected, i.e. mandible cuticle-forming epithelium (around and including the molar area) and gastrolith-forming epithelium, at four molt stages, inter-molt, early pre-molt, late pre-molt and post-molt. RNA was extracted using the TRI reagent (Sigma-Aldrich, St. Louis, MO). Progression of the molt cycle was monitored daily by measuring the gastrolith molt mineralization index (MMI), which was demonstrated to be correlative to molt stages and hormonal titers as described previously [20]. MMI values for each molt stage were as follow: inter-molt, MMI = 0; early pre-molt, MMI 0–0.04; and late pre-molt MMI 0.08-ecdysis. Post-molt animals were harvested on the day following ecdysis. Seven samples were produced from the mandible cuticle-forming epithelium taken from a pool of animals in inter-molt (sampled from three animals), a pool of animals in early pre-molt (sampled from three animals), a pool of animals (sampled from two animals) and two single animals in late pre-molt and two single animals in post-molt. Twelve samples were produced from the gastrolith-forming epithelium taken from two single animals in inter-molt, four single animals in early pre-molt, three single animals in late pre-molt and three single animals in post-molt.

### Construction of a reference transcriptome

A reference transcriptome was constructed by sequencing four sets of samples:

(1) A set of 19 barcoded RNA samples from mandible cuticle-forming epithelium and gastrolith-forming epithelium obtained as described above were sequenced using single-end 50 bp sequencing on a HiSeq2000 apparatus (Illumina, San Diego, CA) at the Applied Genomics Institute (Udine, Italy). For each sample, duplicate reads were removed using CLC Genomics Workbench 6.51 (CLC Bio, Aarhus, Denmark), resulting in a 30% reduction in the number of reads. (2) Pooled RNA extracted from the mandible cuticle-forming epithelium, the carapace cuticle and the gastrolith-forming epithelium. The RNA was sequenced using paired-end 100 bp stretches on one lane of a HiSeq2000 apparatus as described above. (3) A collection of 276,399 reads sequenced using 454 technology, as previously described [19]. (4) A collection of 11,496 expressed sequence tags (ESTs) achieved through Sanger sequencing as previously described [3]. Prior to assembly, CLC Genomics Workbench 6.51 was used to trim remaining trueseq adaptors and low quality base calls from the Illumina and 454 reads. In order to give the Sanger ESTs a higher weight in the assembly compared to the Illumina and 454 reads, each EST was computationally represented 12 times. *De novo* assembly of all sequences was carried out in CLC using default parameters, excluding contigs shorter than 200 bp. Long rows of Ns that were artificially produced by CLC in poor mapping regions between paired reads were shortened to 5 Ns. All reads from sample 1 above were mapped back onto the contigs using CLC, and contigs to which at least one read was mapped were submitted to a second round of assembly in CAP3 [21]. CAP3 Parameters were set to enable mild reduction of redundancy in the transcriptome (-c 12-o 30-p 75-s 500-y 250-z 2), otherwise assembly may result in incorrect joining of gene homologs and splice variants forming artificial chimeras. The result was used in the subsequent analyses as the final molt-related transcriptomic library.

### Differential expression analysis

Single-end 50 bp reads of mandible cuticle- and gastrolith-forming epithelium samples in different molting stages (see (1) above) were aligned to the above transcriptomic library using the alpha-version of STAR 2.3 software [22]. By default, if a read is mapped to multiple locations in the reference sequence with the same best score, STAR randomly chooses one of them as the "primary alignment". However, in our dataset this strategy resulted in only 48% uniquely mapped reads per sample (on average). Moreover, in each sample, an average of 27% reads were mapped to up to 20 loci. As explained above, further reduction of transcriptome redundancy may trade-off with incorrect assemblies, therefore, we eventually used the STAR "—outSAMprimaryFlag AllBestScore" parameter to mark all best-score alignments of the same read as "primary". Additional STAR parameters used in the alignment were "—outFilterMismatchNmax 2—outFilterMultimapNmax 20—outSAMstrandField intronMotif—outFilterMatchNmin 45". The Samtools package was subsequently used to filter out all secondary alignments (-F 0x100). Counting the number of aligned reads per contig per sample was performed using the BEDTools "multicov" tool with the—D flag, thus enabling counting of reads that were mapped to more than one contig with the same primary alignment score. Normalization of the raw counts to account for library size and differential expression analysis were performed using the DESeq R package [23]. DESeq nbinome tests were carried out for the following contrasts: (1) Considering mandible cuticle-forming epithelium samples alone, all possible pairwise comparisons between the different molting stages; (2) considering gastrolith-forming epithelium samples alone, all possible pairwise comparisons between the different molting stages.

### Molt-related binary expression patterns analysis

To study molt-related gene expression, binary expression pattern analysis was developed. Lists of contigs sharing the same binary pattern of expression were constructed for each pattern. Separate pattern lists were generated for each tissue, i.e. lists of contigs for the gastrolith-forming epithelium samples and lists of contigs for the mandible cuticle-forming epithelium samples. The data used for analysis were obtained from the differential expression analysis results (S2 Dataset) as described above. A binary pattern was built in accordance with the quantity of reads which were aligned to the samples in each molt stage, as compared to the other stages. Each stage can either be at a high quantity of reads stage (i.e. significantly different from low quantity of reads stage(s), with a positive fold change) or at a low quantity of reads stage (i.e. significantly different from high quantity of reads stage(s), with a negative fold change). Comparisons were made between each one of the four different molt stages (inter-molt, early pre-molt, late pre-molt and post-molt), yielding a total of sixteen binary patterns. Cutoffs were a *p*-value <0.05 in the pair-wise comparisons and a minimum of 10^3^ reads in the high quantity of reads stage(s). The minimum was set as 10^3^ reads in order to retain only those results that that are more likely to bear biological meaning. Based on the above quantitative ‘filter’, it is important to note that the absolute transcript level of contigs sharing a binary pattern might be different meaning that our approach is qualitative, describing relative differences.

**Fig 1:**
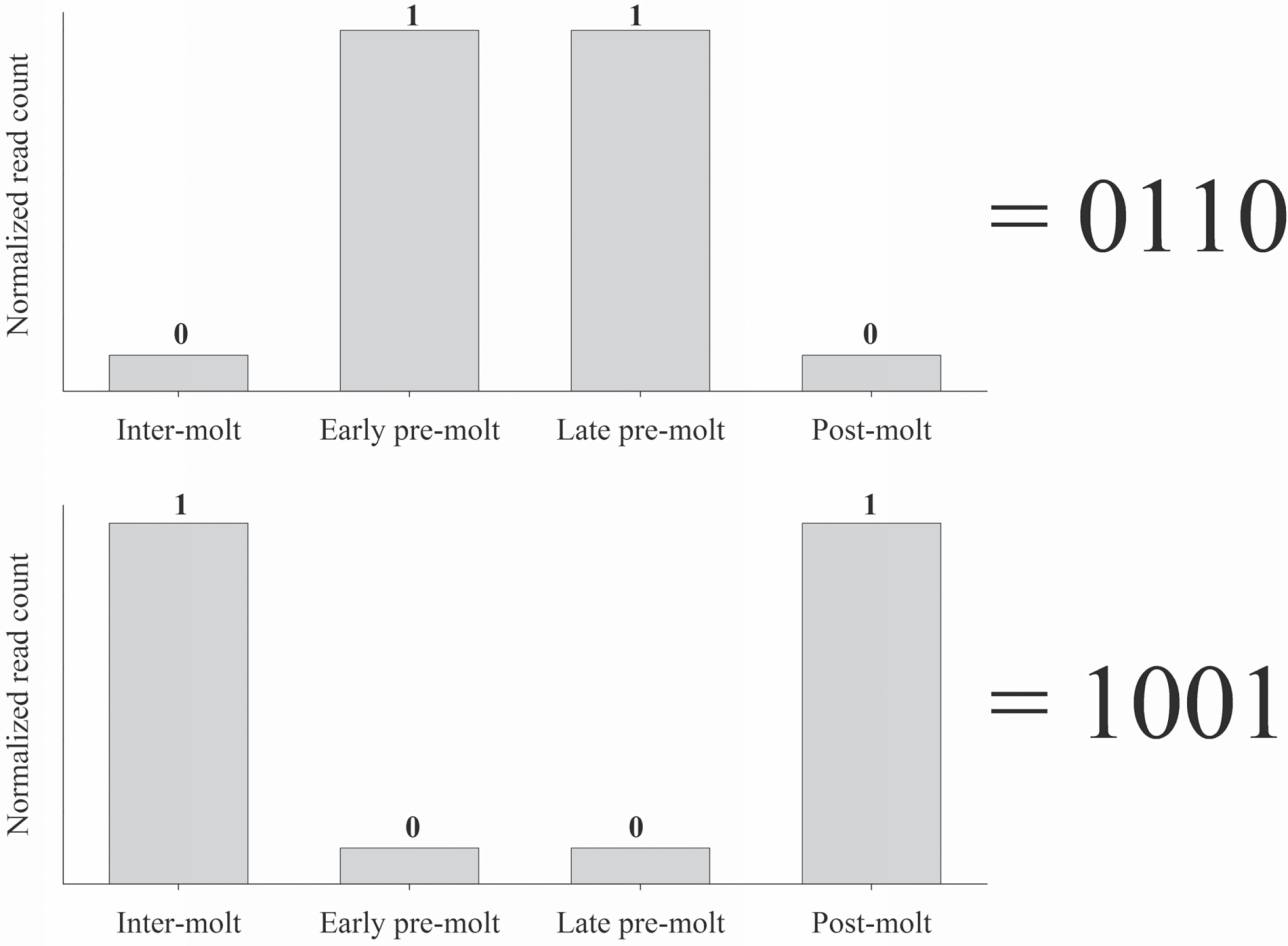
Two representative examples of the binary patterns code used in our study. Top graph—a 0110 pattern in which the quantity of reads in early and late pre-molt is significantly higher, as compared to inter-molt and post-molt (*p*-value <0.05). Bottom graph—a 1001 pattern in which there is a significant increase of reads in inter-molt and post-molt, as compared to early and late pre-molt (*p*-value <0.05). The number 1 in the code represents a high number of reads as compared to a low number of reads, represented with 0. High quantity of reads have a minimum of 10^3^ reads.

For better representation of each pattern, a binary code was created. The binary code represents the quantity of reads at each molt stage. In the code, 0 = low quantity of reads stage, while 1 = high quantity of reads stage. Since four molt stages are present in our transcriptomic library, the binary code for each pattern contains four positions, with each position representing a molt stage, enabling a temporal view. Specifically, position one represents read quantity (0 or 1) during inter-molt, position two represents read quantity (0 or 1) during early pre-molt, position three represents read quantity (0 or 1) during late pre-molt and position four represents read quantity (0 or 1) during post-molt. For further clarity, the use of this binary code is demonstrated in Fig 1, depicting two representative examples (0110 and 1001). This makes our binary code a comprehensive tool to be used throughout the article.

### Annotation and enrichment tests

The Blast2GO software suite [24, 25] was used to predict transcript function and assign Gene Ontology terms [26, 27]. Blast2GO parameters included BLASTX against the GenBank non-redundant (nr) database hosted by the National Center for Biotechnology Information (NCBI) (http://www.ncbi.nlm.nih.gov/), with an e-value threshold, of 1e–6, with retrieval of 3 blast hits for each transcript. Mapping and annotation were performed using default parameters. To enhance transcript function prediction, Annex (annotation augmentation) and an Interpro scan were performed. Combined graphs were calculated with a minimum of 1000 (~10% of the total annotated sequences) per node filter. Enrichment analysis tests were performed using the Blast2GO program with the following parameters set: One-tailed test and double Id's. The primary cut-off was set at a FDR (false discovery rate) < 0.05. When no results were obtained, the test was repeated with a cut-off *p*-value < 0.05 and no FDR cut-off. All results were further reduced to the most specific terms. An enzyme number was assigned to each contig using the Blast2GO program. Enzyme involvement in a metabolic pathway was predicted using the Kyoto Encyclopedia of Genes and Genome (KEGG) server [28].

### Isoforms of chitin metabolism-related genes

We searched for isoforms in our transcriptomic library of the enzymes chitin synthase, uridylyltransferase, chitinase, chitin deacetylase, glucosamine 6-P synthase and glucosamine-6-P deaminase. We considered a contig to be an isoform if the contig was annotated based on highly significant BLAST results (E-value <1*10^–40^) with a similar function. Regarding enzymes in which isoforms were found, isoforms showing a binary pattern were chosen for further research. When several isoforms showed a binary pattern the most significant isoform was chosen for further research (based on BLAST results, Mapping quality, presence of an open reading frame and total read count) and selected as the representative isoform throughout the entire study. In order to approve the choice of the representative isoform, a similar pattern-sharing chitinase isoform was studied *in vitro*.

### Real-time RT-PCR

Animals were induced for molting and RNA was extracted as described above. RNA was extracted from the exoskeletal tissues (mandible cuticle-forming epithelium and gastrolith-forming epithelium) and from the non-skeletal tissues (hepatopancreas and muscle) during each of the molting cycle stages (inter-molt, n = 5; early pre-molt, n = 6; late pre-molt n = 6; post-molt, n = 6). First-strand cDNA was synthesized by means of a reverse transcriptase reaction using the Quanta BioSciences qScript cDNA Synthesis Kit (Gaithersburg, MD) with 1 mg of total RNA. Relative quantification of transcript levels was determined using Roche Diagnostics FastStart Universal Probe Master mix (Basel, Switzerland) and Roche Universal Probe Library probes. The following primers and probes were used: for chitin synthase, qsynthase- F: 5'- TCGTTGACTTCGATGGTCTTC -3' and qsynthase R: 5'- TCCACCATGGCCAACATTAT -3', Probe #84; for chitinase, qchitinase F: 5'- GGCAATGCTCGACTCCTG -3' and qchitinase R: 5'- TTATCTCCGGCACGTCGTA -3', Probe #84; for chitinase2, qchitinase2 F: 5'- GCGTAAGGAATTTAACAAGTACGG-3' and qchitinase2 R: 5'- GCGGTTTCAACAGTGGATTT -3', Probe #89; for chitin deacetylase, qdeacetylase F: 5'- CGCTCCCACTCGTCATAACT -3' and qdeacetylase R: 5'- TGCTATCCACTCCATCAGTCA -3', Probe 50#; for glucosamine 6-P synthase, qGFAT F: 5'- GCGAAGACCCTGGATAATCTC- 3' and q qGFAT R: 5'- TGCCCTAATGATTTCCGAAG -3', Probe 113#; for glucosamine-6-P deaminase, qisomerase F: 5'- GCATCTTCATCACATAACATAATCG- 3' and q qisomerase R: 5'- TGTGGACAGTGTCAGCCTTC -3', Probe 89#; *C*. *quadricarinatus* 18S (accession no. AF235966), which served as a normalizing gene, was also quantified by means of real-time RT PCR using the primers, qcq18S F: 5'- GGTGCATGCCCGTTCTTA -3' and qcq18S R: 5'-TCGTTCGTTATCGGAATTAACC -3' with the above-mentioned mix and Universal Probe Library Probe #22. Reactions were performed with the ABI Prism7300 Sequence Detection System, Applied Biosystems, (Foster City, CA). All cited transcript levels are relative to muscle tissue transcript levels.

Statistical analysis was performed using non-parametric tests as follows: For relative transcript levels between the molt stages, the Kruskal-Wallis rank sum test was performed followed by multiple pair-wise comparisons using the Wilcoxon rank sum test. Differences were considered statistically significant when p-values < 0.05.

## Results

### The transcriptomic library construction

Next-generation sequencing of the 19 barcoded RNA samples yielded the following average number of reads for each of the selected tissues and molt stages. For the mandible cuticle-forming epithelium samples, the following numbers of reads were obtained: Inter-molt 1.04x10^7^ reads (n = 1); early pre-molt 1.03x10^7^ reads (n = 1); late pre-molt 1.3x10^7^ reads (n = 3, SE = 9.57*10^5^), post-molt 1.16x10^7^ reads (n = 2, SE = 3.22x10^5^). For the gastrolith-forming epithelium samples, the following numbers of reads were obtained: Inter-molt 1.17x10^7^ reads (n = 2, SE = 1.2x10^6^); early pre-molt 7.17x10^6^ reads (n = 4, SE = 5.43x10^5^); late pre-molt 7.468x10^6^ reads (n = 3, SE = 1x10^6^); post-molt 9.04x10^6^ reads (n = 3, SE = 2.44x10^5^). The ample representation of each tissue and molt stage is evident from the similarly high number of reads extracted from each tissue. The total number of reads reached was approximately 1.8*10^8^. The initial assembly resulted in a total of 63,351 contigs, a value that was reduced to 62,242 after filtering out those reads that were not back-mapped. The second round of assembly realized by CAP3 resulted in 2,936 contigs and 53,554 singlets. The contig length per number of contigs (in bp) is shown in S1 Fig. Blast2GO analysis yielded a total of 10,998 annotated sequences from the 56,480 sequences that were submitted. The most prominent GO terms assigned to each GO level are shown in S2 Fig.

### Abundance of binary patterns

**Fig 2:**
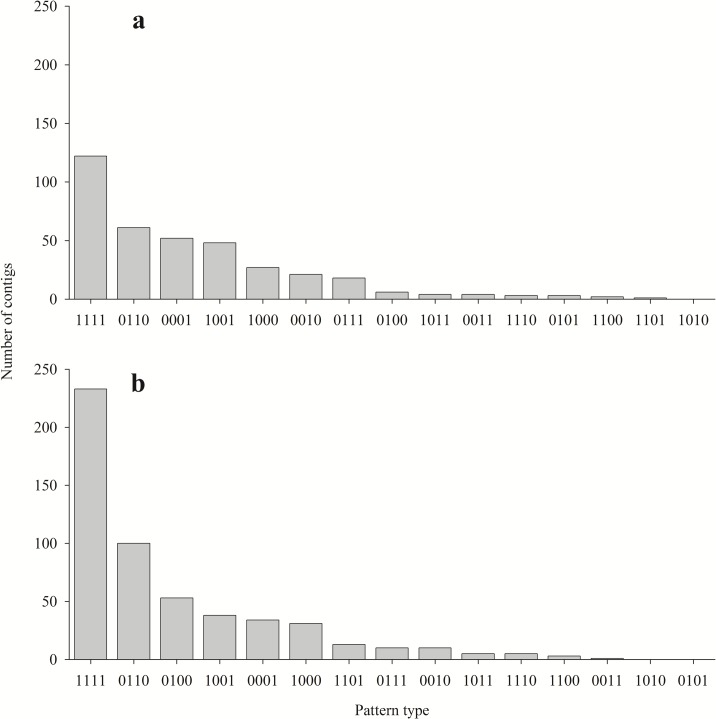
Number of contigs in each binary pattern. (a) Gastrolith-forming epithelium samples and (b) mandible cuticle-forming epithelium samples. Patterns are arranged by abundance, from most to least abundant. A representative example of the binary pattern code is shown in Fig 1.

The above described next-generation sequencing effort, with its considerable representation of all selected tissues and molt stages, enabled testing the abundance of molt-related gene expression patterns using the binary patterning approach (Fig 2). Binary patterns 1111 and 0110 were found to be most abundant in both gastrolith- and mandible cuticle-forming epithelium, although a decrease (approximately 0.5) in abundance in the gastrolith-forming epithelium, as compared to the mandible cuticle-forming epithelium, was seen. Molt-related pattern 1001 was the fourth most abundant value noted in both tissues. The third most abundant pattern differed in each tissue, with 0001 being noted in the gastrolith-forming epithelium (Fig 2A) and 0100 dominating in the mandible cuticle-forming epithelium (Fig 2B). The least abundant patterns, including 0101, 1010, 1101, 1100 and 0011, were similar in both tissues. Pattern 0000 is not shown, even though it accounts for approximately half of the contigs in the library (approximately 30,000). This group includes contigs that contain only few mapped reads in all samples collected from each molting stage.

### Enrichment tests of the binary patterns

**Fig 3:**
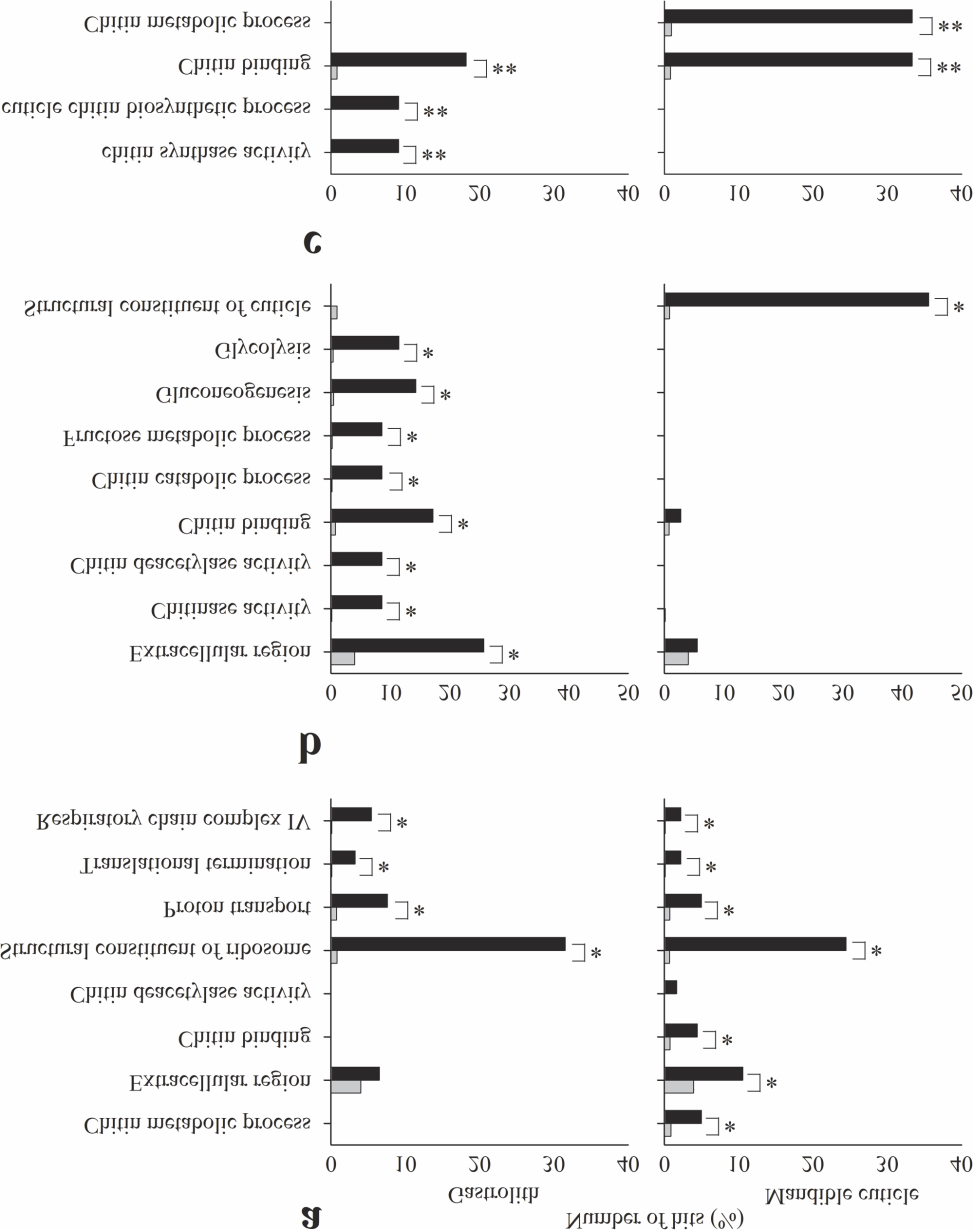
Enrichment analysis test results for different tissues. Enrichment analysis test results of contigs in patterns 1111, 0110 and 0111 (a, b, and c, respectively) from the gastrolith- (left) and mandible cuticle-forming (right) epithelia. Black bars represent the observed number of hits in the sample, while grey bars represent the expected number of hits if the sample was chosen randomly. Significant differences between observed and expected number of hits are indicated by * (FDR <0.05) or ** (*p*-value <0.05).

To study typical annotated gene patterns related to the selected tissues and molt stages, enrichment tests were conducted for the lists of contigs found in each binary pattern (Fig 3). The results showed that several assigned GO terms are enriched in different patterns, when comparing mandible cuticle- and gastrolith-forming epithelia.

In the case of pattern 1111 (high numbers of reads at all molt stages; Fig 3A), gastrolith- and mandible cuticle-forming epithelial transcripts were found to be enriched in assignments to housekeeping gene-related GO terms, such as structural constituent of ribosome (FDR <0.05). The mandible cuticle-forming epithelial transcripts were additionally enriched in assignments to chitin metabolism-related GO terms, such as chitin metabolic process, and extracellular matrix (FDR <0.05) (Fig 3A).

Examination of pattern 0110 (high numbers of reads during pre-molt stages; Fig 3B) revealed that gastrolith-forming epithelial transcripts were enriched in assignments to chitin breakdown-related GO terms, such as chitin catabolic process-, extracellular matrix- and simple sugars metabolism-related GO terms, such as glycolysis (FDR <0.05). The mandible cuticle transcripts were enriched solely in entries assigned a structural constituent of cuticle GO term (FDR <0.05).

Regarding pattern 0111 (high numbers of reads during pre-molt and post-molt stages; Fig 3C), the gastrolith-forming epithelial transcripts were enriched in assignments to chitin synthesis-related GO terms, such as chitin synthase activity (*p*-value <0.05). Transcripts from both tissues were enriched in assignments to chitin-binding GO terms (*p*-value <0.05). The mandible cuticle-forming epithelium was found to be enriched in assignments to chitin metabolic process-related GO terms (*p*-value <0.05).

### Binary patterns of chitin metabolism-related genes

**Fig 4:**
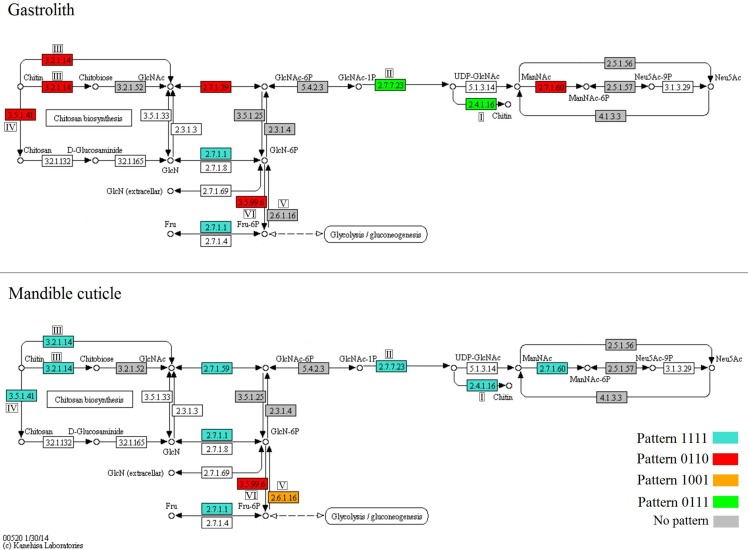
Duplicates of the amino sugar metabolism pathway, modified from KEGG. The upper panel shows gene expression patterns in the gastrolith-forming epithelium, while the lower panel shows gene expression patterns in the mandible cuticle-forming epithelium. Enzymes that were found in the transcriptomic library are highlighted in blue for pattern 1111, red for pattern 0110, orange for pattern 1001, green for pattern 0111 and grey for enzymes that did not show any pattern. Enzymes discussed in the text are marked as I for chitin synthase, II for uridylyltransferase, III for chitinase, IV for chitin deacetylase, V for GlcN-6P synthase and VI for GlcN-6P deaminase.

**Fig 5:**
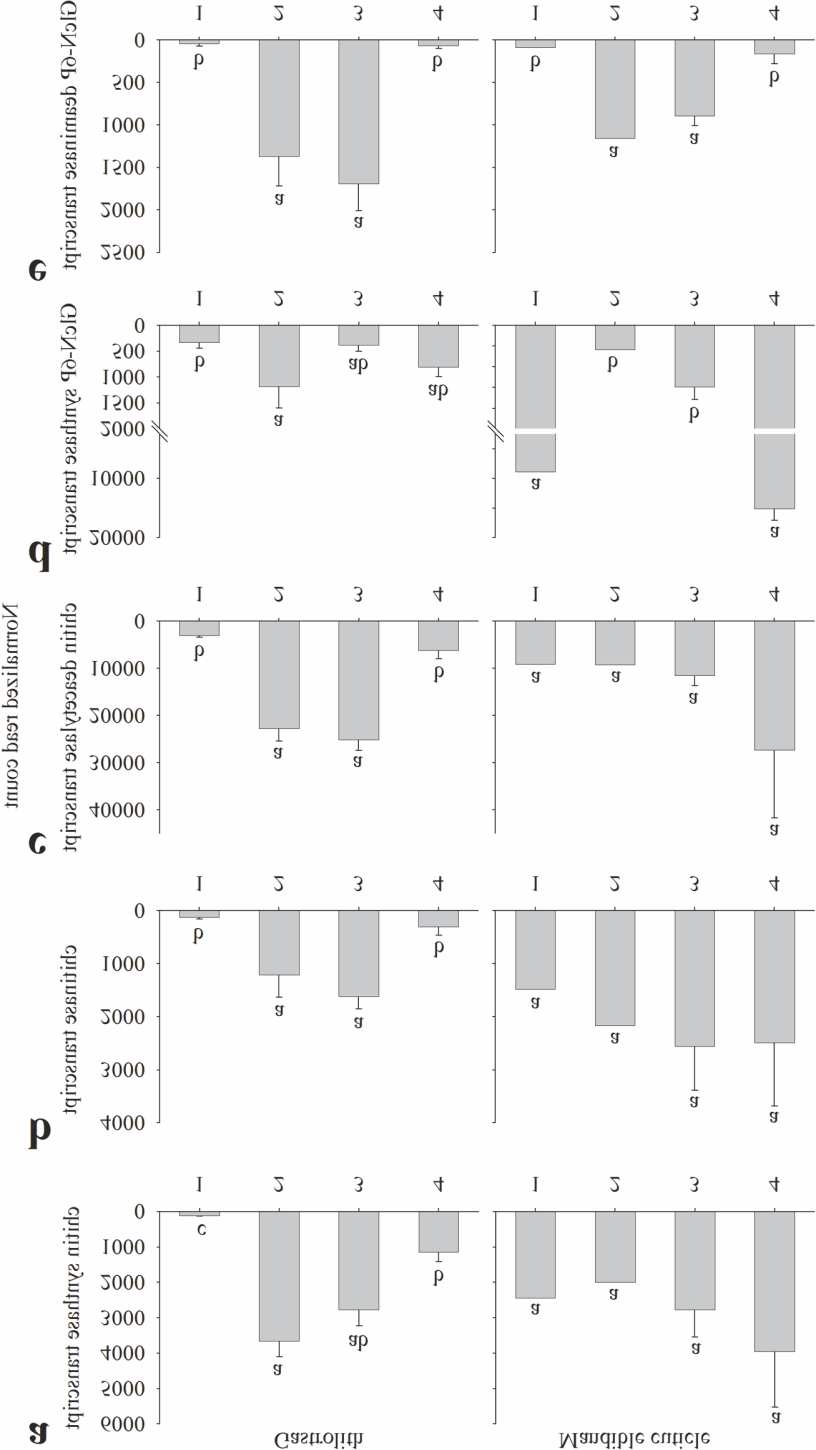
Normalized read count of key chitin metabolism-related genes transcripts. Read count of key chitin metabolism-related genes transcripts from the gastrolith-forming epithelium (left) and the mandible cuticle-forming epithelium (right). Numbers on the X axis represent the four molt stages, 1 inter-molt (pool of animals, n = 1), 2 early pre-molt (pool of animals, n = 1), 3 late pre-molt (two single animals and one pool, n = 3) and 4 post-molt (all single animals, n = 2). Presented transcripts are (a) chitin synthase, (b) chitinase, (c) chitin deacetylase, (d) GlcN-6P synthase and (e) GlcN-6P deaminase. Letters represent statistical groups which are significantly different (*p*-value <0.05), error bars represent standard error.

**Fig 6:**
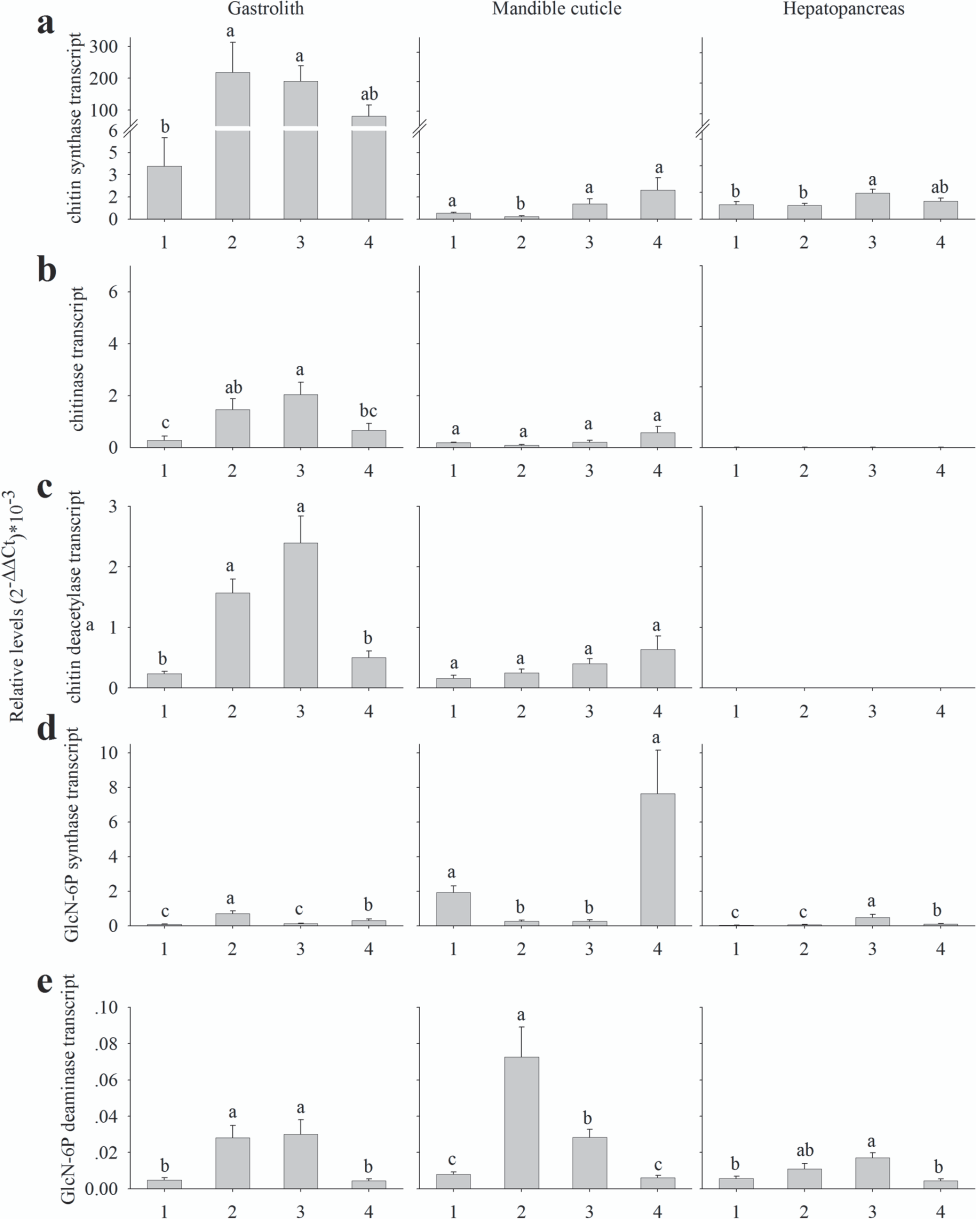
Relative levels of key chitin metabolism-related genes transcript. Relative levels of key chitin metabolism-related genes transcript from the gastrolith-forming epithelium (left), the mandible cuticle-forming epithelium (middle) and the hepatopancreas (right), as determined by qPCR. Numbers on the X axis represents the four molt stages, 1 inter-molt (n = 5), 2 early pre-molt (n = 6), 3 late pre-molt (n = 6) and 4 post-molt (n = 6). Presented transcripts are (a) chitin synthase, (b) chitinase, (c) chitin deacetylase, (d) GlcN-6P synthase and (e) GlcN-6P deaminase. Letters represent statistical groups which are significantly different (*p*-value <0.05), error bars represents standard error.

Since chitin metabolism was found to be prominently enriched in the above described tests, we focused on particular key genes related to main processes related to such metabolism. To follow the expression of relevant genes involved in the amino sugar metabolism pathways (a schematic representation modified from KEGG is presented in Fig 4), normalized read count representations were gathered from the molt-related transcriptomic library (Fig 5) and an *in vitro* confirmation of the values was obtained by real time PCR (Fig 6). Our results show that in general, chitin synthesis- and degradation-related genes in the gastrolith-forming epithelium follow molt-related binary patterns, while in the mandible cuticle-forming epithelium, these genes follow a molt-independent binary pattern.

Two key enzymes in the metabolic pathway of chitin synthesis are chitin synthase (Fig 4I) and uridylyltransferase (Fig 4II). Genes encoding for these enzymes were expressed in the gastrolith-forming epithelium in a molt-related binary pattern, 0111, these enzymes were expressed in a molt-independent binary pattern, 1111, in the mandible cuticle-forming epithelium. The binary expression pattern of chitin synthase (Fig 5A) was mostly, but not fully, confirmed *in vitro* (Fig 6A). In the gastrolith-forming epithelium, post-molt expression was higher than inter-molt expression, although the difference was not statistically significant (Fig 6A, left). In the mandible cuticle-forming epithelium, early pre-molt expression was significantly lower than in the other three stages (Fig 6A, middle). These genes had no probable isoforms in our transcriptomic library.

Chitinase (Fig 4III) and chitin deacetylase (Fig 4IV) are two key enzymes involved in chitin degradation. The studied isoforms of genes encoding for these enzymes were expressed in the gastrolith-forming epithelium in a molt-related pattern, 0110 (Fig 5B and 5C, left). Inversely, in the mandible cuticle-forming epithelium, these enzymes were found to be expressed in a molt-independent binary pattern, 1111 (Fig 5B and 5C, middle). The binary expression pattern of chitinase (Fig 5B) was generally confirmed *in vitro* (Fig 6B) with one exception. In the gastrolith-forming epithelium, post-molt expression was lower than early pre-molt, although the difference was not statistically significant (Fig 6B, left). Chitinase had a total of twenty two other probable isoforms. It is important to note that isoform discovery with no genomic data is a daunting process and some of our probable isoforms might be false positives (e.g. a gene divided to several contigs). Normalized read count of these isoforms is shown in S3 Fig. Thirteen isoforms had a binary pattern in at least one of the forming epithelia. From these isoforms, ten had a 0000 binary pattern (S3L–S3U Fig) and two had similar patterns as our studied isoform (S3C and S4A Fig) in both gastrolith- and mandible cuticle-forming epithelia. The binary pattern of the chitinase isoform studied *in vitro* (named chitinase2) was generally confirmed with one exception. In the mandible cuticle-forming epithelium, post-molt expression was significantly higher than in early pre-molt and inter-molt (S4B Fig). The binary expression pattern of chitin deacetylase (Fig 5C) was fully confirmed *in vitro* (Fig 6C) in both tissues. Chitin deacetylase had a total of four additional probable isoforms. Normalized read count of these isoforms is shown in S5 Fig. Two isoforms shared the exact similar binary patterns of our studied isoform in both gastrolith- and mandible cuticle-forming epithelia (S5A and S5B Fig). Two isoforms had a 0000 binary pattern in most cases (S5C and S5D Fig).

Glucosamine-6-phosphate synthase (GlcN-6P synthase) (Fig 4V) is involved in the utilization of fructose-6-phosphate for amino sugars synthesis. No clear binary pattern was suggested for this enzyme in the gastrolith-forming epithelium (Fig 5D), while in the mandible cuticle-forming epithelium, GlcN-6P synthase was expressed in a molt-related binary pattern, 1001. The binary expression pattern of GlcN-6P synthase in the mandible cuticle (Fig 5D) was confirmed *in vitro* (Fig 6D). In the gastrolith-forming epithelium, some differences in the relative expression were seen between the *in silica* and qPCR results (Figs 5D and6D, left). This gene had no probable isoforms in our transcriptomic library.

Glucosamine-6-P deaminase (GlcN-6P deaminase) (Fig 4VI) is involved in the creation of fructose-6-phosphate from amino sugars. The encoding gene was expressed in a molt-related binary pattern, 0110, in both gastrolith- and mandible cuticle-forming epithelia. The binary expression pattern of GlcN-6P deaminase (Fig 5E) was *in vitro* confirmed in both tissues (Fig 6E). This gene had no probable isoforms in our transcriptomic library.

### Chitin metabolism-related genes expression in the hepatopancreas

The hepatopancreas, responsible for nutrient storage and energy homeostasis in crustaceans, served as a control non-skeletal metabolic tissue in this study. qPCR expression of the key chitin metabolism-related genes listed above suggested possible involvement of the hepatopancreas in chitin metabolism. Three of the tested transcripts revealed a molt-related pattern in the hepatopancase. Chitin synthase gene expression was up-regulated during late pre-molt, as compared to inter-molt and early pre-molt (Fig 6A, left), GlcN-6P synthase gene expression was up-regulated during late pre-molt and post-molt, as compared to inter-molt and early pre-molt (Fig 6D, left), while GlcN-6P deaminase gene expression was upregulated during late pre-molt, as compared to inter-molt and post-molt (Fig 6E, left). Conversely, probable isoforms transcripts of the chitin degradation-related genes chitinase and chitin deacetylase, were not detectable in the hepatopancreatic qPCR assay (Fig 6B and 6C, left, S4B Fig left.).

## Discussion

A transcriptomic library was constructed to study complex gene expression patterns in exoskeletal tissues during distinctive stages of the molt cycle. When approaching such vast quantities of complex data, a comprehensive approach must be considered. Gene expression clustering [29] is a common approach for gene expression studies [30], however, for our objectives this strategy could only provide a partial picture since it compares gene expression relative to only a single point of reference (corresponding to a single molt stage in our case). As such, a comparative study of genes thought to be expressed in multiple different patterns in each molt stage of two exoskeletal tissues, as conducted in the current study, thus requires a different approach. Accordingly, we developed binary expression patterning of the molt-related transcriptomic library. The obvious weakness of such an approach lies in the fact that contigs with a non-binary expression pattern (a ‘grey area’ pattern) are not represented in the study. However, since the crustacean molt cycle is characterized by extreme physiological changes, our binary patterning approach is well representative of typical molt stage-related gene expression patterns. Indeed, the most abundant patterns found in the library are patterns concomitant with the molt cycle events, such as 1111, corresponding to a house-keeping gene-like pattern related to basic metabolism, or 0110 and 1001, which are directly related to molting activity. On the other hand, patterns that were less represented, such as 0101 and 1100, do not seem to be concomitant with molt cycle events.

Enrichment tests are powerful tools for the analysis of RNA expression by focusing on a given group of genes that share a biological feature [31]. Analyzing the different binary patterns lists of contigs combined with enrichment tests, enabled the identification of the case-study of the present research, chitin metabolism. Chitin metabolism-related GO terms were abundant in the enrichment test of the above binary pattern-based lists of contigs. The following discussion thus focuses on three key chitin metabolic processes which emerged from the above enrichment test, chitin synthesis, chitin breakdown and the junction between the metabolism of simple sugars and amino sugars.

Chitin synthesis is catalyzed by the enzyme chitin synthase (EC 2.4.1.16). The activated sugar donor in the reaction is UDP-N-acetylglucosamine. Uridylyltransferase (EC 2.7.7.23) catalyzes the creation of UDP-N- acetylglucosamine [32]. In the gastrolith-forming epithelium, chitin synthase and uridylyltransferase were highly expressed during the pre-molt and post-molt stages (0111 pattern), reflecting a strong relation with molt cycle events. Since the gastrolith undergoes a rapid buildup during pre-molt [10], a sharp increase in chitin synthesis is expected. During post-molt, the gastrolith of the next molting cycle starts to develop [33], possibly involving buildup of chitinous layers, although in the gastrolith-forming epithelium, it remains to be demonstrated that this series of events also occurs. On the other hand, in the mandible cuticle-forming epithelium, chitin synthase and uridylyltransferase transcripts presented a binary expression pattern independent of the molt cycle (the 1111 pattern). A slight contradiction was found in our qPCR results, suggesting that there is a decrease in chitin synthase transcript expression during early pre-molt. In early pre-molt, a non chitinous cuticular layer, the epicuitcle, is formed, while in late pre-molt and post-molt, the innermost chitinous cuticular layers, the exocuticle and endocuticle, are formed [34].This might explain the decrease in chitin synthase expression during early pre-molt. Previous studies on chitin synthase expression in crustaceans conducted on the whiteleg shrimp *Litopenaeus vannamei* [17] showed a similar decrease in early pre-molt, as compared to late pre-molt and post-molt. However, these earlier findings do not coincide with ours suggesting that inter-molt expression is down-regulated and that post-molt expression was significantly higher than in late pre-molt. A different study on a presumable chitin synthesis-related enzyme in the purple shore crab *Hemigrapsus nudus* found that activity was high during early inter-molt, with the decrease seen in late inter-molt [35] providing support for our inter-molt expression results. In addition, the molt-related expression pattern of chitin synthase in the hepatopancraes suggests its possible involvement in the production of chitin during molting.

Chitin breakdown is catalyzed through two possible pathways, namely breakdown of the (1→4)-β-glycosidic bond via hydrolysis through the actions of chitinase (EC 3.2.1.14) and β-N-acetylglucosaminidases (EC 3.2.1.52) or via deacetylation catalyzed by chitin deacetylase (EC 3.5.1.41) [36]. The studied isoforms of chitin breakdown-related genes in the gastrolith-forming epithelium were highly expressed during pre-molt stages (0110 pattern) in a strong molt cycle-related manner. The gastrolith-forming epithelium is solely responsible for gastrolith buildup as degradation of the gastrolith during post-molt occurs in the stomach [10]. However, chitin degradation and modification may play roles in the remodeling of chitinous scaffolds during their synthesis [37], providing a possible explanation for the observed expression pattern. Little is known of chitin breakdown-related genes in the gastrolith-forming epithelium, excluding a mention of a gastrolith protein involved in matrix assembly which was found to have a polysaccharide deacetylase domain [38]. In the mandible cuticle-forming epithelium, the studied isoforms of chitin breakdown-related genes were expressed independently of the molt cycle (1111 pattern). Pre-molt chitin degradation activity might be related to the degradation of the old cuticle at the same time as new chitinous cuticular layers are being synthesized [39]. Since none of the chitinous structures of the mandible cuticle undergo degradation during post-molt or inter-molt, the expression of chitin degradation-related genes at these stages might be related to the remodeling process, as discussed above. Similar results regarding the activity of chitinase during post-molt were found in the Antarctic krill *Euphausia superba* [40], the fiddler crab *Uca pugilator* [41] and in the expression of a chitinase transcript in the whiteleg shrimp *Litopenaeus vannamei* [17]. Only chitin breakdown-related genes had isoforms in our transcriptomic library. Other studies showed that chitin breakdown-related genes have several isoforms in arthropods [17, 42, 43]. Isoforms showing similar pattern as our studied isoform were well represented in our transcriptomic library for both chitinase and chitin deacetylase. While no expression of these chitinase and chitin deacteylase isoforms was found in the hepatopancreas using qPCR. Other isoforms showing a different pattern to our studied isoform were in general less represented in the transcriptomic library. Therefore our studied isoforms are supposedly active isoforms in the exoskeletal tissues while the other isoforms might be involved in different tissues. Previous studies have already found that chitin breakdown-active tissues such as the cuticle forming-epithelium and the hepatopancreas have different active isoforms of the enzyme chitinase [17, 43]. A specific study aiming at isoforms of chitin breakdown-related genes in the crayfish *C*. *quadricarinatus* should be conducted in order to achieve a better understanding of this important physiological aspect.

The process by which simple sugars are aminated or deaminated represents a junction between the metabolism of simple sugars, such as glucose, and the metabolism of amino sugars. These amino sugars could serve for the synthesis of chitin building blocks or originate from the degradation of chitin, thus connecting the above metabolic processes. GlcN-6P synthase (EC 2.6.1.16) catalyzes the transfer of an amine group from glutamine to fructose-6-phosphate. GlcN-6P deaminase (EC 3.5.99.6) catalyzes the deamination of glucosamine 6-phosphate to produce fructose-6-phosphate and ammonia [44–46]. In the gastrolith-forming tissue, the GlcN-6P synthase transcript was not expressed in any of the binary patterns considered here, although a significant increase in expression was recorded in early pre-molt and post-molt, as compared to late pre-molt and inter-molt. In contrast, in the mandible cuticle-forming epithelium, GlcN-6P synthase was expressed in non-pre-molt stages (pattern 1001), while GlcN-6P deaminase expression presented a mirror image of the former (0110) in both gastrolith- and mandible cuticle-forming epithelia. Prior studies suggested that in the cuticle of the crayfish *Orconectes obseurus*, N-acetyl glucosamine-6-phosphate utilization for chitin synthesis was mainly noted during pre-molt [16], whereas the use of glucose for chitin synthesis was mainly observed during post-molt [18] In the crayfish *Orconectes sanborni*, glucosamine-6-phosphate synthesis in the cuticle was high during post-molt and in early inter-molt [47]. Integrating our results with the insights obtained in these earlier studies allowed us to hypothesize that during pre-molt stages, glucosamine-6-phosphate (obtained from the breakdown of the old cuticle) is utilized to synthesize chitin or degraded to simple sugars, like glucose. The enrichment test results regarding the 0110 binary pattern suggest a need for glucose during pre-molt in the gastrolith-forming epithelium. In contrast, during post-molt and inter-molt, chitin precursors derived from the degradation of the old cuticle are not available and hence the need for amino sugars is presumably satisfied by *de novo* synthesis. In the hepatopancreas, GlcN-6P synthase and GlcN-6P deaminase showed molt-related expression patterns, suggesting their involvement in amino sugar metabolism during the molt cycle.

Binary patterning of our molt-related transcriptomic library resulted in the description of typical chitin metabolism-related gene expression patterns in two exoskeltal tissues during four molt stages. Since the gastrolith-forming epithelium undergoes major changes during specific stages of the molt cycle, chitin metabolism was predicted to be highly related to this cycle. Despite the fact that the mandible cuticle-forming epithelium undergoes similar major changes during the molt cycle, chitin degradation and synthesis seem to occur independently of molt cycle stages and rather seem to be part of constant molt-independent metabolic processes that should be further investigated. The use of the binary patterning approach described here provided a unique integrative picture of chitin metabolism-related gene activity during the molt cycle. Such binary patterning could be used in the study of gene activity in other complex biological processes in which both temporal and spatial aspects must be simultaneously addressed. Further development of this approach is needed to reach a consensus definition for binary patterning and resolving difficulties concerning ‘grey area’ type expression patterns.

## Supporting Information

S1 Dataset Blast2GO analysis results. A list of all blast 2GO analysis results for every contig including: sequence description, sequence length, number of blast hits, minimum E-value, mean similarity, GO terms, Enzyme assigned and Interpro results. Also included all annotated sequences and annotated sequences from three binary patterns list (1111, 0110 and 0111) from both gastrolith- and mandible cuticle-forming epithelia.
(XLSX)

S2 Dataset Differential expression analysis results. Differential expression results for every contig including; Normalized read count at each sample, fold change between all molt stages in each tissue, log2 fold change between all molt stages in each tissue and statistical results (shown as *p*-value) of the pairwise comparisons between all molt stages in each tissue.
(XLSX)

S1 Fig Distribution of contig lengths in the transcriptomic library. (TIF)

S2 Fig Most prominent GO terms in the transcriptomic library. Most prominent GO terms as calculated by Blast2GO software suite processes. (a) Level 3 of molecular functions, (b) level 2 of cellular components and (c) level 1 of biological processes.
(PDF)

S3 Fig Normalized read count of chitinase isoforms transcripts. Read count of twenty two chitinase isoforms transcripts found in our transcriptomic library, from the gastrolith-forming epithelium (left) and the mandible cuticle-forming epithelium (right). Numbers on the X axis represent the four molt stages, 1 inter-molt (pool of animals, n = 1), 2 early pre-molt (pool of animals, n = 1), 3 late pre-molt (two single animals and one pool, n = 3) and 4 post-molt (all single animals, n = 2). Letters represent statistical groups which are significantly different (*p*-value <0.05), error bars represent standard error.
(TIF)

S4 Fig Normalized read count and relative levels of chitinase2 transcript. Read count (a) from the gastrolith-forming epithelium (left) and the mandible cuticle-forming epithelium (right). Relative levels (b) from the gastrolith-forming epithelium (left), the mandible cuticle-forming epithelium (middle) and the hepatopancreas (right), as determined by qPCR. Numbers on the X axis represent the four molt stages, 1 inter-molt (pool of animals, n = 1), 2 early pre-molt (pool of animals, n = 1), 3 late pre-molt (two single animals and one pool, n = 3) and 4 post-molt (all single animals, n = 2). Letters represent statistical groups which are significantly different (*p*-value <0.05), error bars represent standard error.
(TIF)

S5 Fig Normalized read count of chitin deacetylase isoforms transcripts. Read count of four chitin deacetylase isoforms transcripts found in our transcriptomic library, from the gastrolith-forming epithelium (left) and the mandible cuticle-forming epithelium (right). Numbers on the X axis represent the four molt stages, 1 inter-molt (pool of animals, n = 1), 2 early pre-molt (pool of animals, n = 1), 3 late pre-molt (two single animals and one pool, n = 3) and 4 post-molt (all single animals, n = 2). Letters represent statistical groups which are significantly different (*p*-value <0.05), error bars represent standard error.
(TIF)
