# Correction: Binary Gene Expression Patterning of the Molt Cycle: The Case of Chitin Metabolism

**DOI:** 10.1371/journal.pone.0130787

**Published:** 2015-06-12

**Authors:** Shai Abehsera, Lilah Glazer, Jenny Tynyakov, Inbar Plaschkes, Vered Chalifa-Caspi, Isam Khalaila, Eliahu D. Aflalo, Amir Sagi

Fig 3 and Fig 5 in this article appear upside-down. Please see the correct figures here.

**Fig 3:**
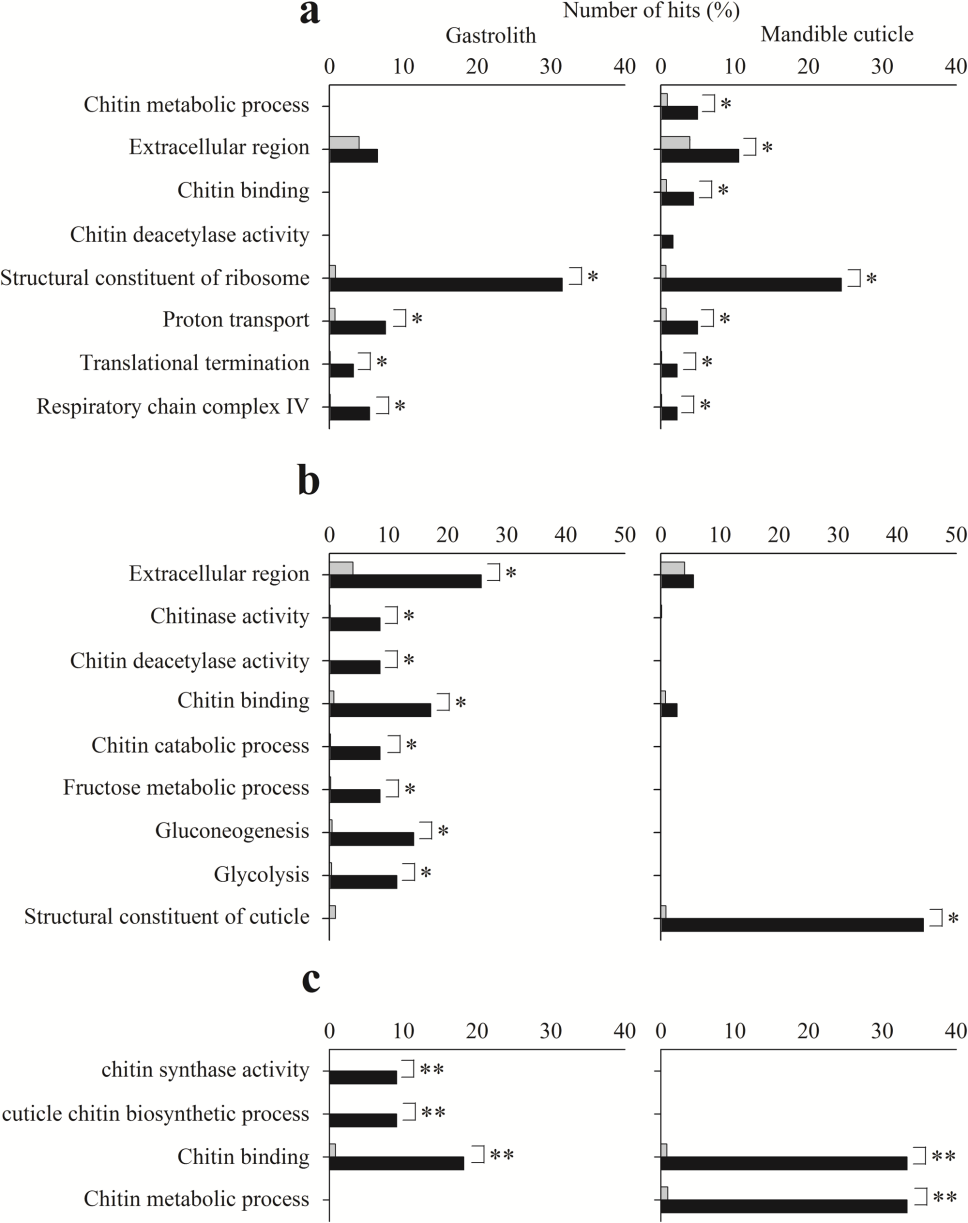
Enrichment analysis test results for different tissues. Enrichment analysis test results of contigs in patterns 1111, 0110 and 0111 (a, b, and c, respectively) from the gastrolith- (left) and mandible cuticle-forming (right) epithelia. Black bars represent the observed number of hits in the sample, while grey bars represent the expected number of hits if the sample was chosen randomly. Significant differences between observed and expected number of hits are indicated by * (FDR <0.05) or ** (*p*-value <0.05).

**Fig 5:**
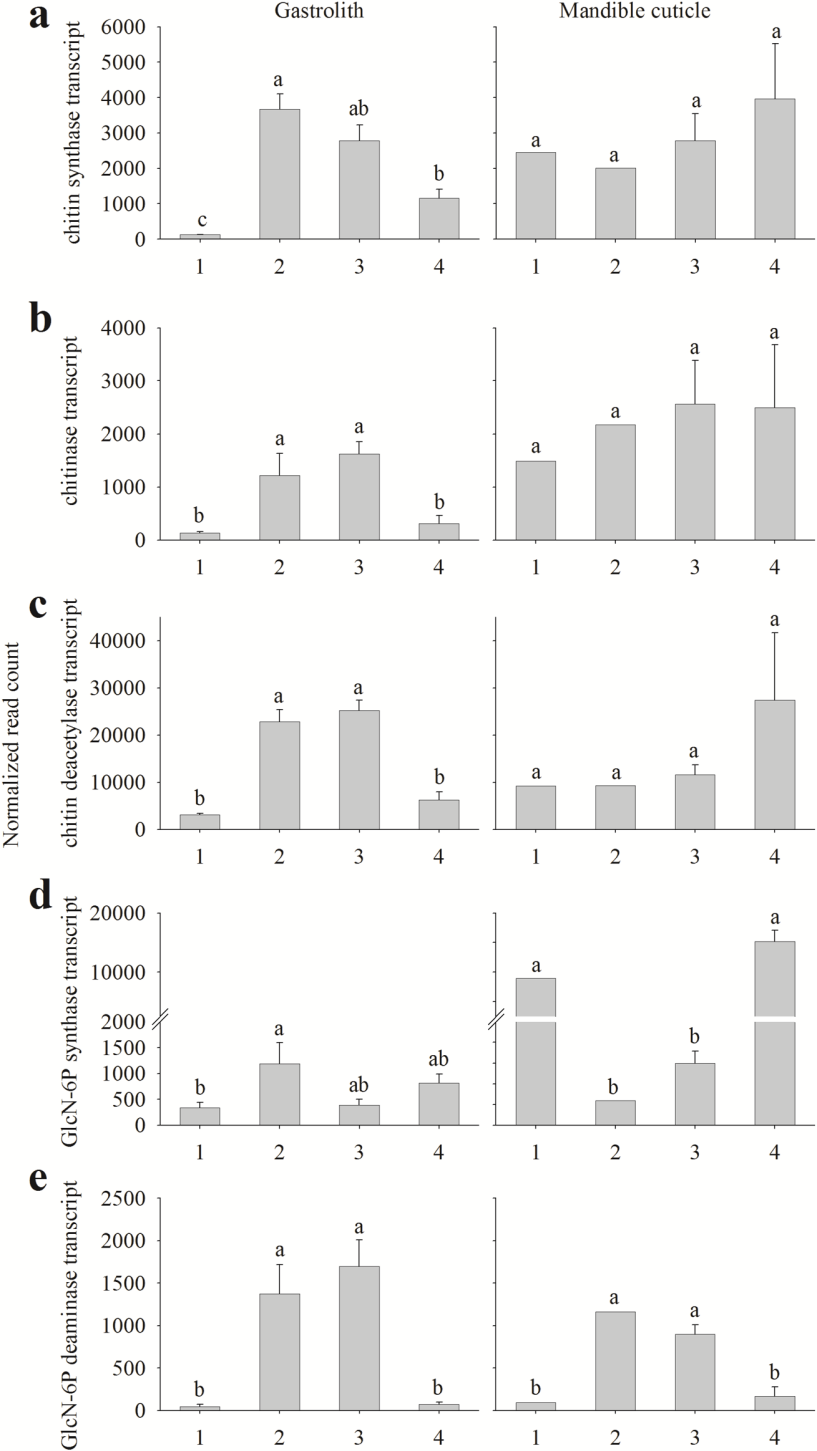
Normalized read count of key chitin metabolism-related genes transcripts. Read count of key chitin metabolism-related genes transcripts from the gastrolith-forming epithelium (left) and the mandible cuticle-forming epithelium (right). Numbers on the X axis represent the four molt stages, 1 inter-molt (pool of animals, n = 1), 2 early pre-molt (pool of animals, n = 1), 3 late pre-molt (two single animals and one pool, n = 3) and 4 post-molt (all single animals, n = 2). Presented transcripts are (a) chitin synthase, (b) chitinase, (c) chitin deacetylase, (d) GlcN-6P synthase and (e) GlcN-6P deaminase. Letters represent statistical groups which are significantly different (*p*-value <0.05), error bars represent standard error.

## References

[1] Abehsera S, Glazer L, Tynyakov J, Plaschkes I, Chalifa-Caspi V, Khalaila I, et al (2015) Binary Gene Expression Patterning of the Molt Cycle: The Case of Chitin Metabolism. PLoS ONE 10(4): e0122602 doi:10.1371/journal.pone.0122602 25919476 10.1371/journal.pone.0122602 PMC4412622

