# Supplementary figures and images for: Binary Gene Expression Patterning of the Molt Cycle: The Case of Chitin Metabolism
Source: PLoS One. 2015 Apr 28;10(4):e0122602. doi: 10.1371/journal.pone.0122602 (PMC4412622; doi:10.1371/journal.pone.0122602)

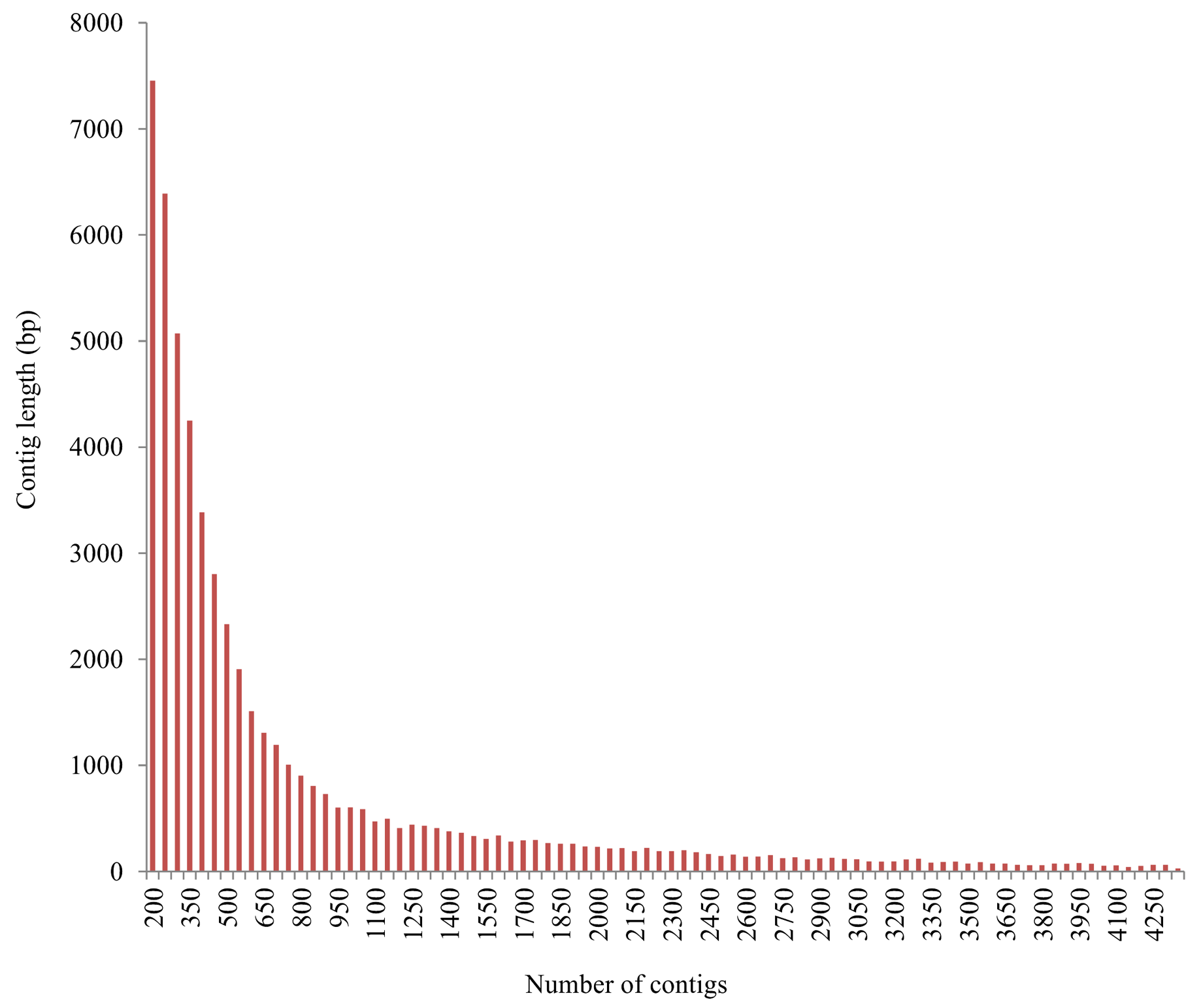

Supplement: S1 Fig — (TIF) [file pone.0122602.s003.tif]

**a**

## Molecular function

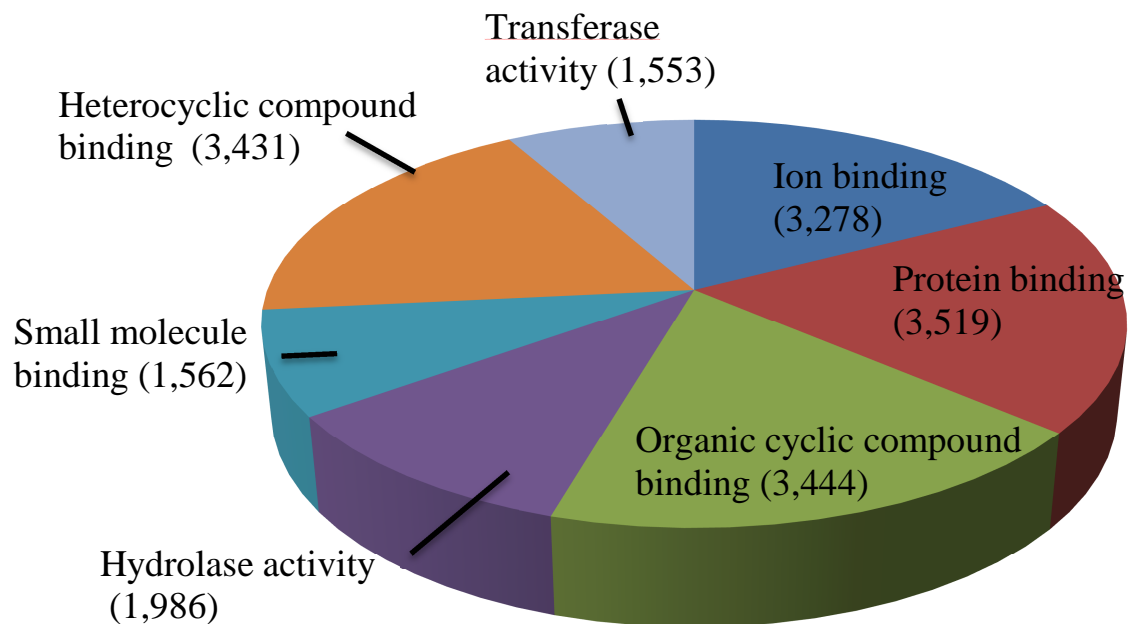

**b**

## Cellular component

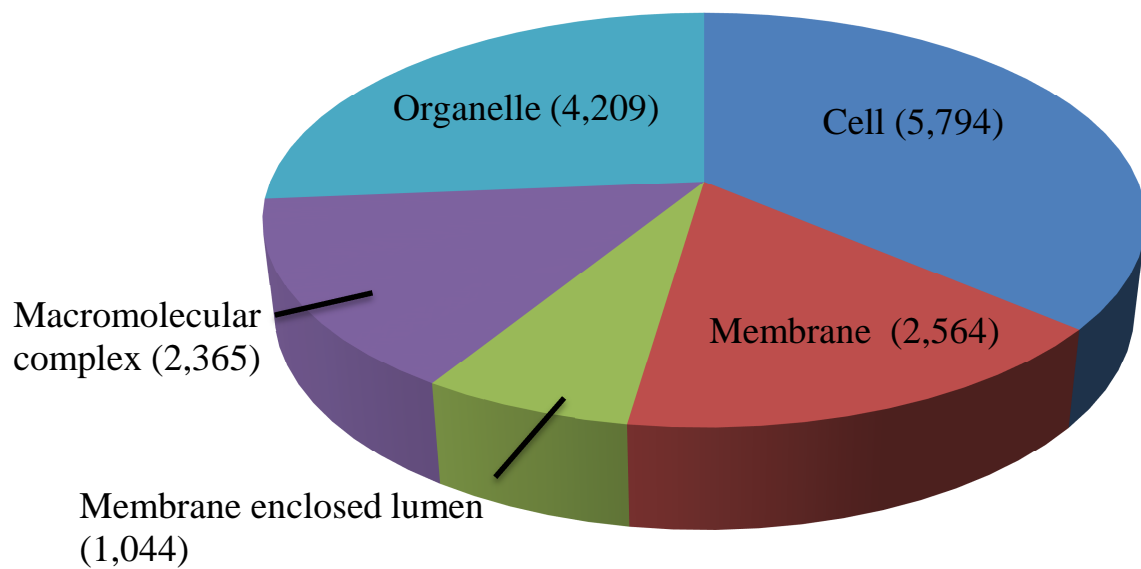

**c**

## Biological process

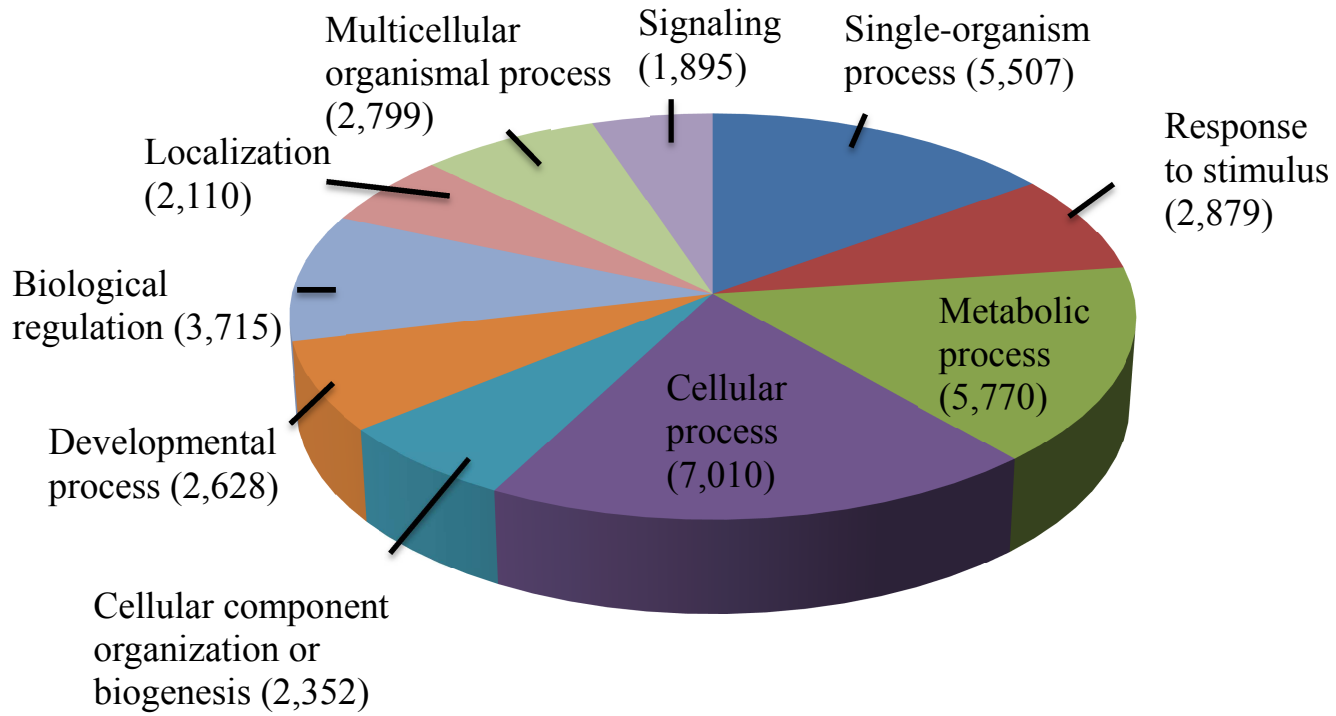

Supplement: S2 Fig — Most prominent GO terms as calculated by Blast2GO software suite processes. (a) Level 3 of molecular functions, (b) level 2 of cellular components and (c) level 1 of biological processes. (PDF) [file pone.0122602.s004.pdf]

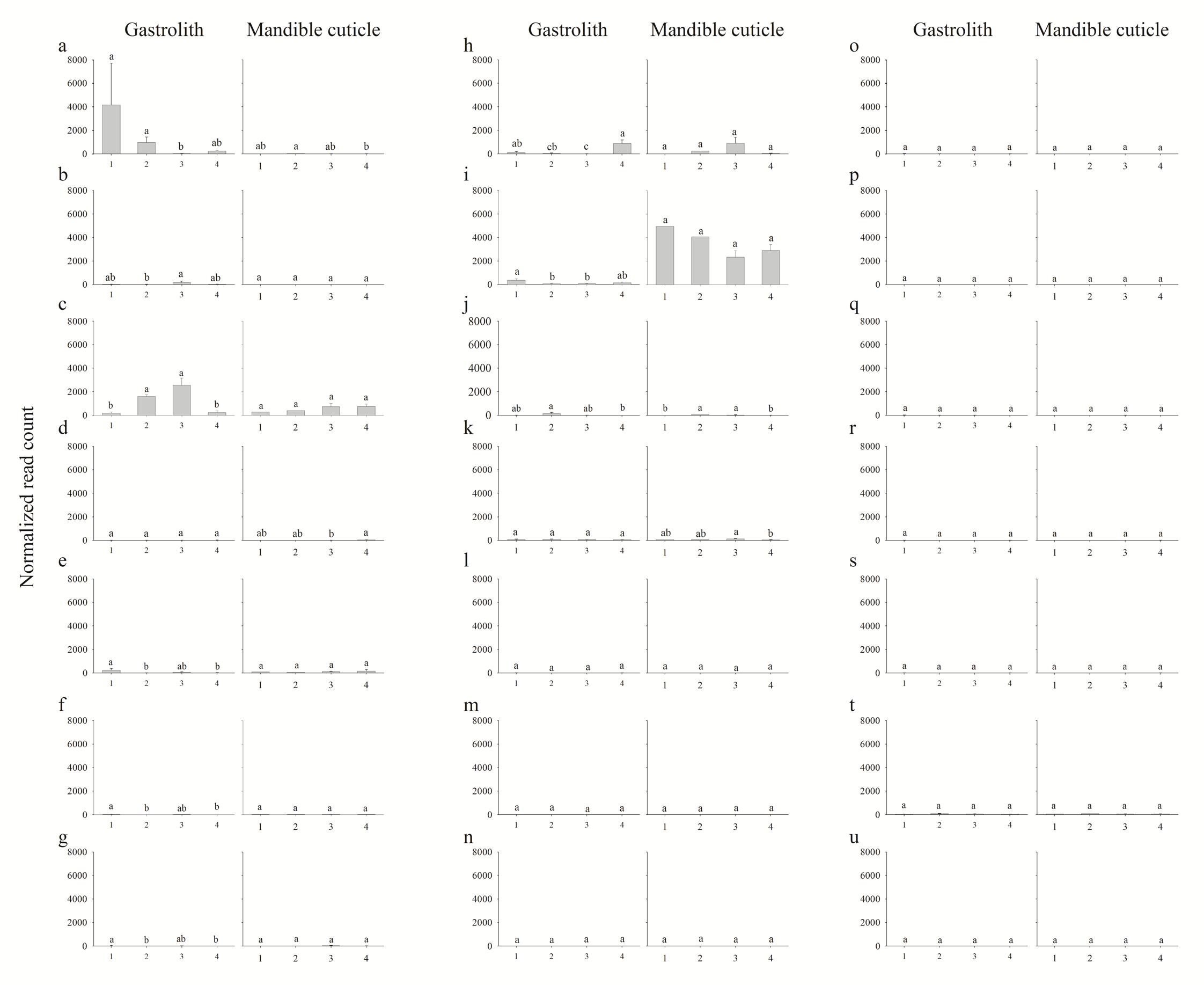

Supplement: S3 Fig — Read count of twenty two chitinase isoforms transcripts found in our transcriptomic library, from the gastrolith-forming epithelium (left) and the mandible cuticle-forming epithelium (right). Numbers on the X axis represent the four molt stages, 1 inter-molt (pool of animals, n = 1), 2 early pre-molt (pool of animals, n = 1), 3 late pre-molt (two single animals and one pool, n = 3) and 4 post-molt (all single animals, n = 2). Letters represent statistical groups which are significantly different (p-value <0.05), error bars represent standard error. (TIF) [file pone.0122602.s005.tif]

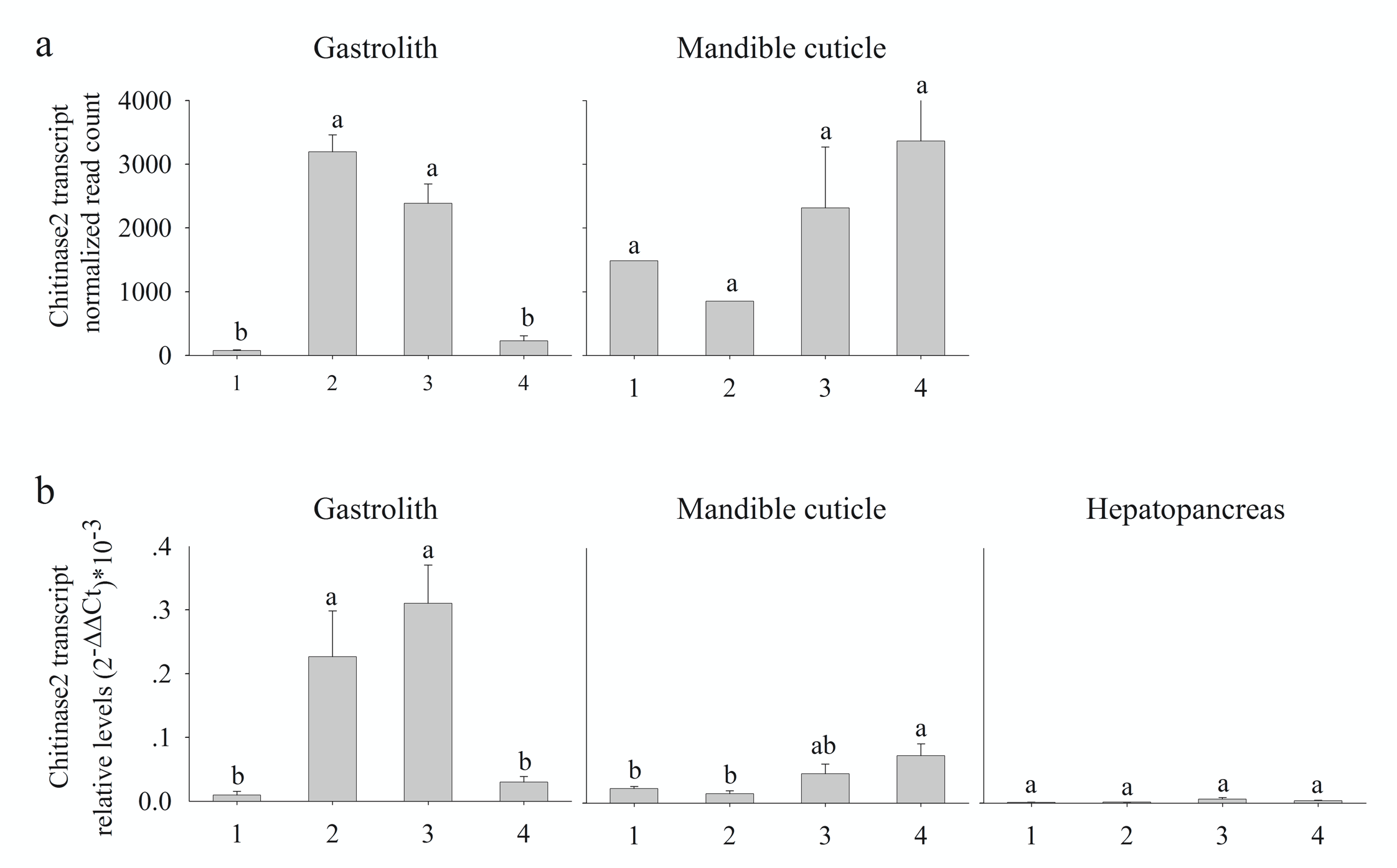

Supplement: S4 Fig — Read count (a) from the gastrolith-forming epithelium (left) and the mandible cuticle-forming epithelium (right). Relative levels (b) from the gastrolith-forming epithelium (left), the mandible cuticle-forming epithelium (middle) and the hepatopancreas (right), as determined by qPCR. Numbers on the X axis represent the four molt stages, 1 inter-molt (pool of animals, n = 1), 2 early pre-molt (pool of animals, n = 1), 3 late pre-molt (two single animals and one pool, n = 3) and 4 post-molt (all single animals, n = 2). Letters represent statistical groups which are significantly different (p-value <0.05), error bars represent standard error. (TIF) [file pone.0122602.s006.tif]

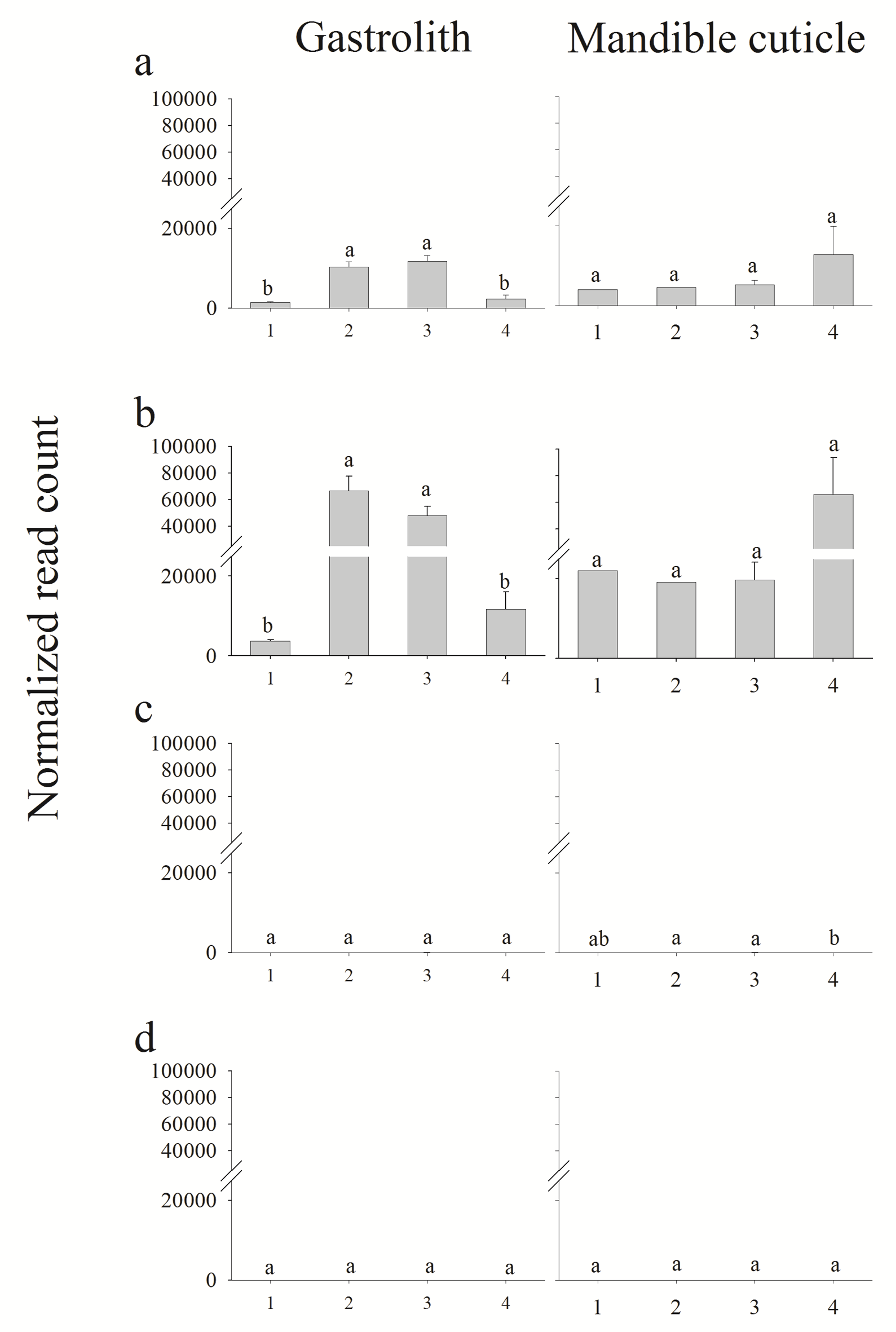

Supplement: S5 Fig — Read count of four chitin deacetylase isoforms transcripts found in our transcriptomic library, from the gastrolith-forming epithelium (left) and the mandible cuticle-forming epithelium (right). Numbers on the X axis represent the four molt stages, 1 inter-molt (pool of animals, n = 1), 2 early pre-molt (pool of animals, n = 1), 3 late pre-molt (two single animals and one pool, n = 3) and 4 post-molt (all single animals, n = 2). Letters represent statistical groups which are significantly different (p-value <0.05), error bars represent standard error. (TIF) [file pone.0122602.s007.tif]
